# Development and validation of a sepsis diagnostic scoring model for neonates with suspected sepsis

**DOI:** 10.3389/fped.2022.1004727

**Published:** 2022-10-06

**Authors:** Rozeta Sokou, Georgios Ioakeimidis, Daniele Piovani, Stavroula Parastatidou, Aikaterini Konstantinidi, Andreas G. Tsantes, Maria Lampridou, Dimitra Houhoula, Nicoletta Iacovidou, Styliani Kokoris, Aristeidis G. Vaiopoulos, Argyri Gialeraki, Petros Kopterides, Stefanos Bonovas, Argirios E. Tsantes

**Affiliations:** ^1^ Neonatal Intensive Care Unit, “Agios Panteleimon” General Hospital of Nikea, Piraeus, Greece; ^2^ Department of Biomedical Sciences, Humanitas University, Milan, Italy; ^3^ IRCCS Humanitas Research Hospital, Milan, Italy; ^4^ Laboratory of Haematology and Blood Bank Unit, “Attiko” Hospital, School of Medicine, National and Kapodistrian University of Athens, Athens, Greece; ^5^ Neonatal Department, National and Kapodistrian University of Athens, Aretaieio Hospital, Athens, Greece; ^6^ Intensive Care Unit, Excela Health Westmoreland Hospital, Greensburg, PA, United States

**Keywords:** thromboelastometry, prediction model, neonatal sepsis, septic shock, neonatal sepsis diagnostic score

## Abstract

**Background:**

We aimed to develop and validate a diagnostic model for sepsis among neonates evaluated for suspected sepsis, by incorporating thromboelastometry parameters, maternal/neonatal risk factors, clinical signs/symptoms and laboratory results.

**Methods:**

This retrospective cohort study included 291 neonates with presumed sepsis, hospitalized in a NICU, from 07/2014 to 07/2021. Laboratory tests were obtained on disease onset and prior to initiating antibiotic therapy. Τhromboelastometry extrinsically activated (EXTEM) assay was performed simultaneously and Tοllner and nSOFA scores were calculated. Sepsis diagnosis was the outcome variable. A 10-fold cross-validation least absolute shrinkage and selection operator logit regression procedure was applied to derive the final multivariable score. Clinical utility was evaluated by decision curve analysis.

**Results:**

Gestational age, CRP, considerable skin discoloration, liver enlargement, neutrophil left shift, and EXTEM A10, were identified as the strongest predictors and included in the Neonatal Sepsis Diagnostic (NeoSeD) model. NeoSeD score demonstrated excellent discrimination capacity for sepsis and septic shock with an AUC: 0.918 (95% CI, 0.884–0.952) and 0.974 (95% CI, 0.958–0.989) respectively, which was significantly higher compared to Töllner and nSOFA scores.

**Conclusions:**

The NeoSeD score is simple, accurate, practical, and may contribute to a timely diagnosis of sepsis in neonates with suspected sepsis. External validation in multinational cohorts is necessary before clinical application.

## Introduction

Sepsis is a leading cause of morbidity and mortality in neonates, with an estimated worldwide incidence of approximately 22 neonates per 1,000 livebirths and a mortality rate of 11%–19%, depending on the study population and the definition of cases (1). Sepsis is the third most common cause of death during the neonatal period and accounts for one fourth of all neonatal deaths (2).

Early diagnosis of neonatal sepsis is crucial and remains a major challenge for the neonatologists; yet a single respective definition does not exist. Determination of diagnostic criteria for neonatal septicemia is of great importance, as the delay in management of severe bacterial infections could negatively affect the clinical outcome of neonates. The clinical signs of neonatal sepsis are non-specific, especially at early stages, and they are often attributed to non-infectious conditions. As early diagnosis of neonatal sepsis is difficult, due to non-specific symptoms and laboratory tests with limited diagnostic value, empirical and non-judicious use of antibiotics is common practice in Neonatal Intensive Care Units (NICUs) (3). Thus, the development and validation of diagnostic scores for neonatal sepsis is essential.

The course of septic neonates is dramatically aggravated and their prognosis is worsened by the presence of Disseminated Intravascular Coagulation (DIC) or Multi-Organ Dysfunction Syndrome (MODS) (4). Sepsis is frequently complicated with hemostatic derangement due to interaction of coagulation components with inflammatory mediators (5, 6).

Understanding of the multifactorial mechanism of coagulation is considered as a prerequisite for diagnosis of hemostatic disorders, especially in neonates. Rotational thromboelastography (TEG) and thromboelastometry (ROTEM), as point-of-care tests, evaluate global clot formation and dissolution. In adult septic patients, the diagnostic and prognostic role of TEG/ROTEM has been extensively investigated, suggesting that these tests may facilitate diagnosis of clotting abnormalities, and additionally, improve prognosis of sepsis (9). Limited data are available on the role of TEG/ROTEM variables as indicators of neonatal sepsis (7, 8, 10–13).

Diagnosis of sepsis is crucial, yet challenging among ill neonates, as differential diagnosis is extremely complicated in this subpopulation. On the other hand, overdiagnosis of sepsis leads to unnecessary and potentially harmful therapeutic interventions in neonates. To date, no reliable or validated diagnostic score for neonatal sepsis has been established in clinical practice (14, 15). Our aim was to develop and validate a sepsis diagnostic model for hospitalized in NICU neonates undergoing evaluation for sepsis, examining ROTEM parameters in different combinations with maternal/neonatal risk factors, inflammatory response parameters, laboratory tests and physical examination findings.

## Materials and methods

### Participants

This single-center, observational study includes full-term and pre-term neonates with sepsis or suspected sepsis, hospitalized in the NICU of General Hospital of Nikea, Piraeus, Greece, between July 2014 and July 2021. Our hospital is a referral center, and a large proportion of patients are immigrants and low-income native population, with no antenatal care in most cases. The study protocol was designed and conducted in compliance with the Declaration of Helsinki, was approved by the Institutional Review Board of Nikea General Hospital (15 July 2014, 32/3) and all methods were performed in accordance with the relevant guidelines and regulations. Written informed consent has been obtained by parents or guardians of enrolled neonates.

#### Definitions

Early onset sepsis (EOS) was defined as sepsis occurring within the first 72 h of life.

Late onset sepsis (LOS) was defined as sepsis occurring at or after 72 h of life.

Sepsis was defined as the clinical condition characterized by isolation of bacteria or fungi in a blood and/or cerebrospinal fluid (CSF) culture. Neonates >72 h of life with clinical signs of sepsis and a positive urine culture were also considered as septic (15). For diagnosis of Coagulase-negative staphylococci (CoNS) sepsis, 2 consecutive positive blood cultures with the same CoNS bacteria or 1 positive blood culture and simultaneous presence of 2 clinical signs or laboratory data, were required (15).

Neonatal septic shock was defined using the consensus criteria for pediatric sepsis (16, 17), and MODS was defined as concurrent dysfunction of ≥2 systems (18).

#### Inclusion criteria

The presence of clinical signs and/or laboratory data suggestive of sepsis necessitating initiation of antibiotic therapy (15, 17): detailed relevant information provided in the Supplementary data. Absence of exposure to antibiotics for at least 48 h was required for inclusion in the study.

#### Exclusion criteria

Neonates with perinatal asphyxia, major congenital malformation or those transfused with fresh frozen plasma or platelets, were excluded. Additional exclusion criteria were: difficulties during blood sampling, known history of maternal acquired or congenital coagulopathy and treatment interfering with hemostasis.

### Study design

The development and internal validation of the diagnostic score in this study was performed and reported in agreement with the TRIPOD guideline (19).

On the first day (day 1) of sepsis or suspected sepsis, data on demographics, maternal and pregnancy history, and medications received during pregnancy, were recorded. Prior to initiating antibiotic therapy, blood specimens for culture, routine biochemical tests, arterial blood gas, complete blood count, peripheral blood smear and CRP were obtained. Simultaneously, 300 µl of residual arterial blood was analyzed on the ROTEM analyzer (Tem Innovations GmbH, Munich, Germany) using the standard extrinsically activated (EXTEM) assay. ROTEM, which evolved from the original TEG system, is a whole blood viscoelastic, dynamic method for global evaluation of hemostasis. EXTEM assay is activated by recalcification and addition of tissue thromboplastin. It reflects the activity of the extrinsic pathway of coagulation, as generation and formation of the clot mainly depends on coagulation factors VII, X, V, II, and fibrinogen (20). The following EXTEM parameters were measured: Clotting Time (CT, seconds), defined as the time from the beginning of the test to a clot amplitude of 2 mm; Clot Formation Time (CFT, seconds), indicating time from CT until clot amplitude of 20 mm is achieved; Alpha-angle (*α*°), the angle between the baseline and a tangent of the clotting curve at 2 mm; Clot firmness amplitude recorded at 10, 20, and 30 min (A10, A20, and A30); Maximum Clot Firmness (MCF, mm), reflecting the final strength of the clot; Lysis Index at 60 min (LI 60%), defined as the percentage of remaining clot in relation to the MCF following the 60-min observation period after CT; and Maximum Lysis (ML), representing the percent decrease of maximal amplitude over time.

GA, birthweight, delivery mode, and potential maternal risk factors for sepsis [prolonged and/or premature rupture of membranes, fever, chorioamnionitis, positive colonization with Group B Streptococcus (GBS) or history of previous infant with GBS infection and GBS bacteriuria] were recorded.

The evaluated clinical variables included 5-minutes Apgar score, temperature instability, jaundice, cyanosis, edema or sclerema, petechiae, poor peripheral perfusion assed by capillary refill time (CRT, defined as “normal” for CRT <3 s; “impaired” for CRT 3 to 5 s; and “considerably impaired” for CRT > 5 s), change in skin color (evaluated as “normal” for bright pink or pink coloration, “moderate change” for mild or inconstant skin discoloration, “considerable change” for conspicuous and constant change of color towards green grey skin coloration), poor feeding, irritability, seizure, lethargy, gastrointestinal symptoms, vomiting, abdominal distention, hepatomegaly (palpable liver >2 cm below the right costal margin), apnea, tachypnea, need for respiratory support, grunting, tachycardia, bradycardia, mean blood pressure, hypotension, and urine output.

Laboratory data included: blood pH, base deficit, plasma lactate levels, PaO_2_/FIO_2_, hematocrit level, white blood cells, neutrophil count and neutrophil left shift (immature leukocytes present in peripheral blood), platelet count, CRP, serum albumin, serum glutamic oxaloacetic transaminase (SGOT), serum glutamic pyruvic transaminase (SGPT), total bilirubin, direct/indirect bilirubin, and plasma creatinine. The distribution of each candidate continuous variable was inspected. Prior to any analysis, we applied a log10-transformation to serum albumin, white blood cells, CRP, SGOT, SGPT, total bilirubin, direct bilirubin, indirect bilirubin, creatinine, as their distribution was severely right skewed.

Chest radiograph, cerebrospinal fluid culture and urine culture were performed whenever clinically indicated. In these neonates, serial blood samples for sepsis biomarkers were obtained, when deemed necessary, until antibiotic therapy was completed.

In all cases of sepsis or suspected sepsis, Tοllner and Neonatal Sequential Organ Failure Assessment (nSOFA) scores were calculated (21, 22).

### Statistical analysis and model development

We presented the baseline characteristics and ROTEM parameters of the study population as means ± standard deviations (SDs), medians and interquartile ranges (IQRs), or percentages, when appropriate.

Our aim was to build a practical, multivariable diagnostic score for the prompt diagnosis of sepsis in neonates with suspected sepsis, using ROTEM parameters and clinical and biochemical characteristics.

Initially, we investigated the association between ROTEM parameters and sepsis. We assessed multicollinearity by using the non-parametric Spearman correlation test. Most parameters were very highly correlated (*r_S_* > 0.80 and *p* < 0.05). To avoid substantial multicollinearity in our model development, the ROTEM parameter most associated with sepsis in a univariable logistic regression (i.e., highest likelihood ratio chi-squared test), i.e., A10 was retained as the candidate predictor (23). A complete list of all candidate predictors is available in our extended method section (Supplementary data).

The multivariable model was selected and fitted my means of L1-penalized least absolute shrinkage and selection operator (LASSO) logit regression (24–26). Model fitness was checked by using the Hosmer-Lemeshow test which is a goodness-of-fit test comparing observed and expected probabilities into quantiles of linear predictor (27). Missing data did not exceed 2% for any candidate predictor. We considered them as missing at random and we performed a complete-case analysis.

We developed a diagnostic score [Neonatal Sepsis Diagnostic (NeoSeD) score] by converting the *β* coefficient of each predictor in the final model into a weighted score using integer values while preserving monotonicity and simplicity. We reported the full model specifications in a table including the model intercept, as recommended by the TRIPOD guidelines (19). We produced a nomogram plot transforming all possible total point scores into the corresponding absolute risk of sepsis. Finally, we performed a decision curve analysis as recommended by Vickers et al. (28) to assess the clinical utility of the NeoSeD score by quantifying the net benefit when different threshold probabilities for a diagnosis of sepsis were considered (a guide to interpreting decision curve analysis is provided in Supplementary data Appendix S1).

### Internal validity, calibration and performance measures

We selected variables entering the final model through 10-fold cross-validation (CV) LASSO logit regression, a procedure maximizing the internal validity (ie, reproducibility) of the resulting model (19).

We verified the calibration of our final model through a predicted vs. observed probability plot: the calibration belt (29). To check the presence of any difference between the mean predicted probability and the observed proportion of sepsis cases, we also computed the calibration in the large measure (i.e., it should ideally equal zero) and accompanied it with a formal statistical test under the null hypothesis of no difference (19).

The discrimination capacity of our preliminary diagnostic score was assessed through ROC analyses which allowed the calculation of the AUC (19). We tested the ability of the model to generalize to new cases by using 10-fold cross validation. We calculated the discrimination capacity of our model in each of the 10-fold cross validation subsamples and averaged it reporting the mean cross-validated AUC to provide a more realistic measure of model performance. We also calculated the brier score as a measure of diagnostic accuracy (30). The proportion of variance explained by the model on the logit scale was calculated with the McKelvey–Zavoina pseudo *R*^2^, taking values from 0 (i.e., no variance explained) to 1 (i.e., the entire variance is explained) (31).

The net benefit and the area under curve of the NeoSeD score were formally compared with those of the Tollner and those of the nSOFA scores (21, 22, 32). Though the NeoSeD score was developed as a diagnostic tool for sepsis, we also tested its overall performance on septic shock (secondary outcome measure) and on blood culture-confirmed sepsis. More information regarding our methodology can be found in the Supplementary data. All tests were two-sided. The STATA (Stata Corp., College Station, Texas, United States) and R software were used for statistical modeling and analysis. A two-sided *p*-value <0.05 was considered statistically significant.

## Results

### Characteristics of study subjects

The study population consisted of 291 full-term and pre-term neonates with suspected sepsis. We report the baseline characteristics of these neonates in Supplementary Table S1. About half (*N* = 142; 49%) of the included newborns were preterm (<37 weeks of gestation), and one fourth (*N* = 82; 28%) were very preterm (<32 weeks). Half of them had a low birthweight (<2,500 grams; *N* = 145; 50%) and about one third had a very low birthweight (<1,500 grams; *N* = 99; 34%). Out of the 291 studied neonates, 119 (41%) were diagnosed with sepsis: 23 (19%) with EOS, and 96 (81%) with LOS. Among confirmed sepsis cases, the blood culture revealed the presence of Gram negative bacteria in 62 cases (51%), Gram positive spp. in 21 cases (18%), Candida in 12 cases (10%). Of the remaining sepsis cases, 10 neonates (8%) had positive CNS culture and 14 (12%) had positive urine culture. Among septic neonates 61 (51.3%) were diagnosed with septic shock and 18 (15%) died.

### Model development

We sought to identify a multivariable model enabling the early diagnosis of sepsis in a population of neonates with suspected sepsis and developed and internally validated a preliminary diagnostic score using ROTEM parameters and clinical and biochemical characteristics. At the end of the selection process performed by applying 10-fold crossvalidation to a L1-penalized LASSO, 6 variables were identified and included in the final multivariable logit model used for building the Neonatal Sepsis Diagnostic (NeoSeD) Score: Clot firmness amplitude recorded at 10 min (A10, mm), gestational age (GA, weeks), C-reactive protein (CRP, log10 transformation of the value expressed in mg/L) as continuous variables, presence of considerable change in skin color, liver enlargement, and neutrofil left shift. The NeoSeD score was built on 287 neonates with suspected sepsis with complete data. Full model specifications are reported in Table 1.

**Table 1:** Risk of sepsis in term and preterm neonates with suspected sepsis: model specifications of the final multivariable logit model selected by applying 10-fold cross-validation to a L1-penalized least absolute shrinkage and selection operator (LASSO) logit procedure.

Predicting variable	*β* coefficient (95% CI)	*p*-value
A10 (mm)	−0.0309 (–0.0596, –0.0022)	0.035
Considerable change in skin color	+2.887 (+1.922, +3.854)	<0.001
Liver enlargement	+1.472 (+0.3516, +2.592)	0.010
Absence of shift to the left	–1.183 (–1.953, –0.414)	0.003
Gestational age (weeks)	–0.189 (–0.272, –0.106)	<0.001
C-reactive protein (mg/L, expressed in log10)	+1.181 (+0.566, +1.796)	<0.001
*model intercept*	+6.095	–

A10, amplitude recorded at 10 min.

We derived the NeoSeD score by converting the *β* coefficients and intercept into a weighted score. We provided detailed and easy-to-apply instructions to calculate the NeoSeD score for each patient given his/her values of relevant predictors (Table 2).

**Table 2:** Neonatal Sepsis Diagnostic (NeoSeD) score: scoring system.

Baseline patient's characteristics	Points
Amplitude at 10 min (mm)	
For each unit	−10
Considerable change in skin color	+934
Liver enlargement	+476
Absence of shift to the left	−383
Gestational age (weeks)	
For each unit	−61
C-reactive protein (mg/L, log10 transformed)^a^	
For each unit	+382
All patients start with 1972 points	

^a^ A value of C-reactive protein of 100 mg/L should be considered as 2 [ie, log_10_(100)].

#### Example of the derivation of the NeoSeD score for one patient with the following characteristics

A10 = 50 mm (−10 × 50 = −500 points);
Presence of considerable change in skin color (+934 points);
Presence of liver enlargement (+476 points);
Absence of shift to the left (−383 points);
37 weeks of gestational age (−61 × 37 = −2,257 points)
100 ml/L of C-reactive protein (2 × 382 = +764 points);
Each patient starts with 1972 points
This patient score is +1,006 points which, according to the nomogram plot in Figure 3, equals to a very high risk of sepsis (>90%)

### Internal validation and measures of model performance

**Figure 1:**
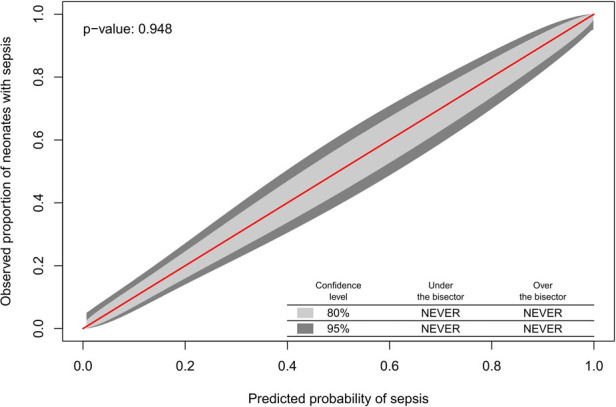
The observed proportion of neonates with sepsis and the probability of sepsis across the entire spectrum of predicted probabilities.

The Hosmer-Lemeshow test indicated good model fit (*p* = 0.60). Internal validity was ensured by the 10-fold cross-validation procedure used during model development. The calibration belt showed optimal calibration (*p* = 0.95), meaning close to perfect agreement between the observed proportion of neonates with sepsis and the probability of sepsis across the entire spectrum of predicted probabilities (Figure 1).

The calibration in the large was equal to 0.00 (*p* = 0.99), meaning that there was no evidence of a significant difference between the mean predicted probability and the observed proportion of sepsis cases. The brier score and the McKelvey–Zavoina pseudo *R*^2^ showed good predictive accuracy and a substantial proportion of the variation in the diagnosis of sepsis explained in our sample (Table 3).

**Table 3:** Performance measures of the Neonatal Sepsis Diagnostic (NeoSeD) score in predicting sepsis (primary outcome),septic shock (secondary outcome) and blood culture-confirmed sepsis (sensitivity analysis) in the study population.

	Sepsis	Septic shock	Blood culture-confirmed sepsis
AUC (95% CI)	0.918 (0.884–0.952)	0.974 (0.958–0.989)	0.868 (0.823–0.914)
CV-mean AUC (95% CI)^a^	0.925 (0.867–0.984)	0.975 (0.933–1.000)	0.874 (0.725–1.000)
Calibration in the large (*p*-value)	0.00 (0.99)	0.00 (0.99)	0.00 (0.99)
Brier score	0.104	0.048	0.132
McKelvey–Zavoina pseudo *R*^2^	0.701	0.776	0.473

CI, confidence interval; CV, cross-validated; AUC, Area under the Curve.

^a^ These AUCs and 95% confidence intervals have been obtained by averaging the AUC in each of the 10 cross-validation samples.

**Figure 2:**
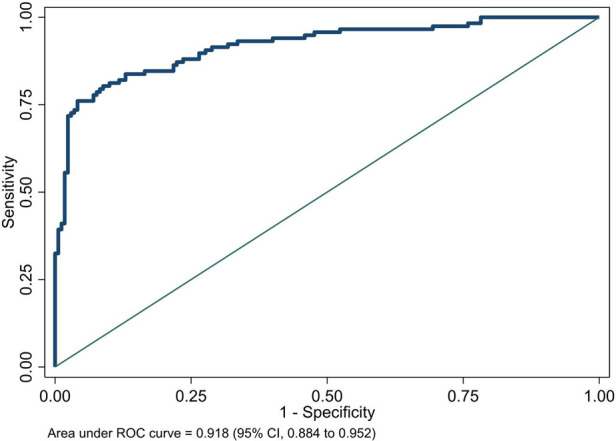
Area under receiver-operating characteristic (ROC) curve of the Neonatal Sepsis Diagnostic (NeoSeD) score.

We plotted the area under the receiver operating characteristic (ROC) curve of the NeoSeD score (Figure 2). We obtained an area under curve (AUC) of 0.918 (95% CI, 0.884–0.952), indicating excellent discrimination capacity.

**Figure 3:**
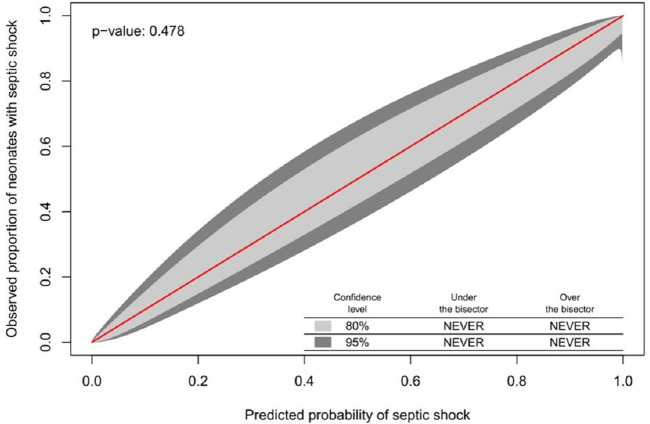
Calibration plot of the Neonatal Sepsis Diagnostic (NeoSeD) score displaying the agreement between the observed proportion of neonates with septic shock (secondary event) and its predicted probability. The red line represents perfect calibration.

**Figure 4:**
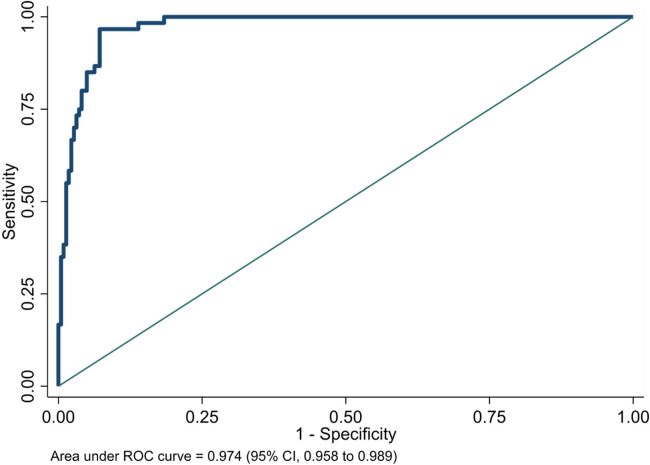
Area under receiver-operating characteristic (ROC) curve of the Neonatal Sepsis Diagnostic (NeoSeD) score: septic shock (secondary outcome).

Although the NeoSeD score was developed for the diagnosis of sepsis (primary outcome), it also showed excellent performances regarding septic shock (secondary outcome). The NeoSeD score showed good calibration (*p* = 0.48, Figure 3) and extremely high discrimination capacity (AUC: 0.974; 95% CI, 0.958–0.989, Figure 4).

**Figure 5:**
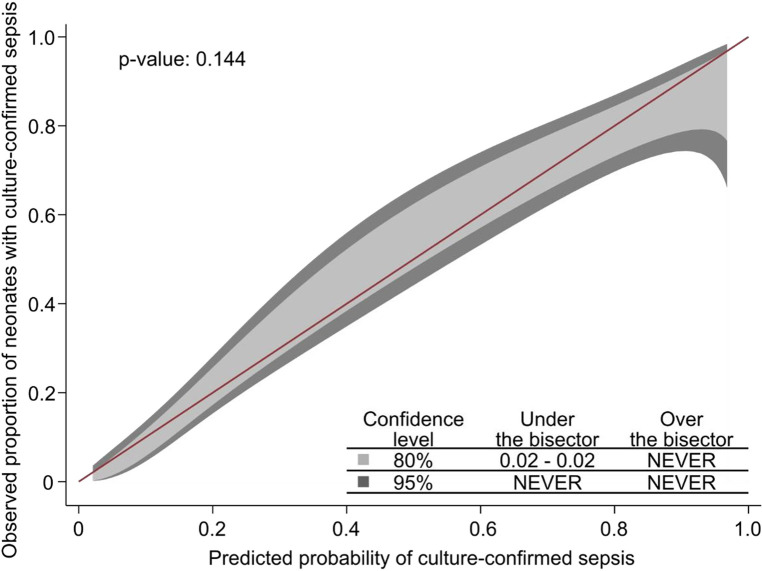
Calibration plot of the Neonatal Sepsis Diagnostic (NeoSeD) score displaying the agreement between the observed proportion of neonates with blood culture-confirmed sepsis (sensitivity analysis) and its predicted probability. The red line represents perfect calibration.

**Figure 6:**
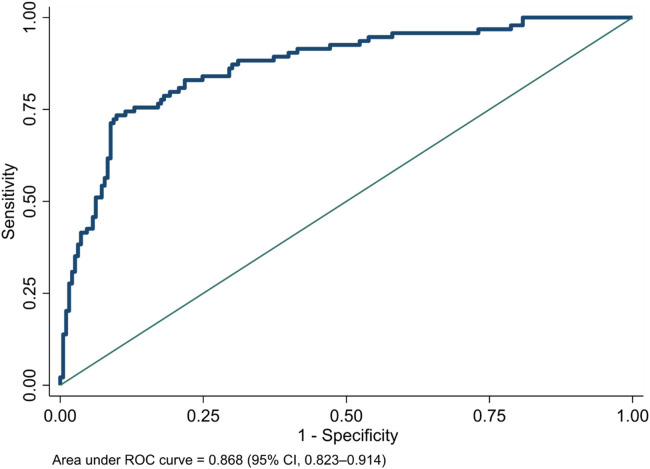
Area under receiver-operating characteristic (ROC) curve of the Neonatal Sepsis Diagnostic (NeoSeD) score: blood culture-confirmed sepsis (sensitivity analysis).

As a sensitivity analysis, we tested the diagnostic value of the NeoSeD score when considering as septic patients only those with a positive blood culture, and it showed good calibration (*p* = 0.14, Figure 5) and high discrimination capacity (AUC: 0.868; 95% CI, 0.823–0.914, Figure 6).

The average cross-validated AUCs for sepsis, septic shock, and blood culture-confirmed sepsis showed a high discrimination capacity (Table 3).

The discrimination capacity of the NeoSeD score was significantly better (ie, higher AUC) than that of the Tollner score in our study population for both sepsis (AUC_Tollner_ for sepsis: 0.843; 95% CI, 0.793–0.893; *p* for the difference <0.001) and septic shock (AUC_Tollner_ for septic shock: 0.948; 95% CI, 0.912–0.984; *p* for the difference = 0.043). Additionally, the discrimination capacity of the NeoSeD score was higher than that of the Neonatal Sequential Organ Failure Assessment (nSOFA) for both sepsis (AUC_nSOFA_ for sepsis: 0.760; 95% CI, 0.703–0.817; *p* for the difference <0.001) and septic shock (AUC_nSOFA_ for septic shock: 0.912; 95% CI, 0.876–0.947; *p* for the difference <0.001).

### Predicted probability of sepsis and septic shock and clinical utility

**Figure 7:**
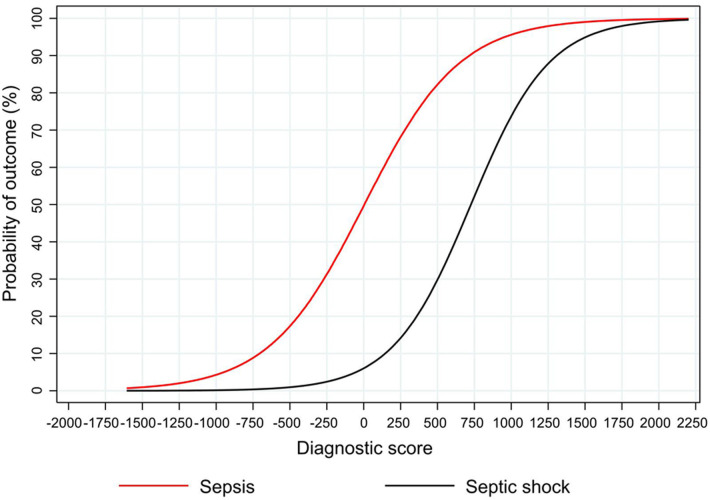
The predicted probability of sepsis and septic shock according to the Neonatal Sepsis Diagnostic (NeoSeD) score.

**Figure 8:**
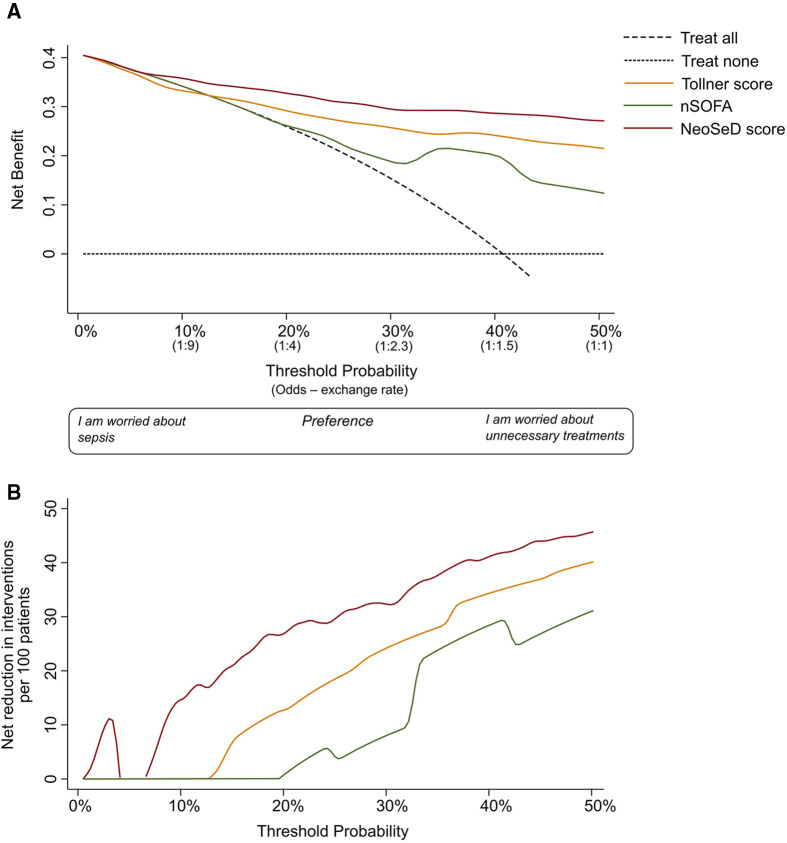
Decision curve analysis for the early detection (before the results of blood culture) of sepsis in critically-ill neonates admitted to the neonatal intensive care unit for the newly developed Neonatal Sepsis Diagnostic (NeoSed) score (red line), the Tollner score (orange line), and the neonatal Sequential Organ Failure Assessment (nSOFA, green line). (**Panel A)** The net benefit (y axis) is a metric representing the benefit of a certain intervention minus its harms, multiplied by an exchange rate. The unit of net benefit is true positives. A net benefit of 0.10, for instance, means “10 true positives for every 100 patients in the target population.” The net benefit is plotted over a range of possible decision thresholds/exchange rates (i.e., individual predicted probabilities given in Figure 3) for the early diagnosis of sepsis (x-axis). The net benefit also incorporates any consequence (i.e., clinical actions) of a diagnosis of sepsis. The net benefit of five different strategies is compared. The two extreme—default—strategies are “treat all” (diagonal dashed line) and “treat none” (orizontal dashed line) meaning enacting clinical actions as if all patients with suspected sepsis were septic (i.e., “treat all”), or as if they were not (i.e., “treat none”). The x-axis can be also renamed as preference: clinicians more worried about the harms of a missed diagnosis of sepsis will adopt thresholds closer to a predicted probability of zero (i.e., left side of the graph), while clinicians more worried about the harms of unnecessary interventions (i.e., on false positives) will adopt higher thresholds (i.e., right side of the graph). The x-axis is also called “exchange rate” which is an odds and represents how many false positives are worth one true positive (i.e., adopting a threshold probability of 10% means that a patient with a predicted probability over 10% will be considered septic—and treated accordingly—and by adopting this classification rule/threshold, one accepts that one true positive is worth nine false positives). Interventions associated with different harms may need the adoption of different thresholds/exchange rates for their applications. The strategy corresponding to the highest net benefit over the largest range of threshold probabilities, should be considered as the best one to adopt. (**Panel B**) In the second graph, net benefit is expressed in terms of “net reduction in unnecessary interventions” in respect to a strategy to “treat all” (i.e., this may be the default strategy for intravenous antibiotics in these patients). This graph is useful to understand whether using the NeoSeD score to identify patients with and without sepsis would help reduce unnecessary empiric antibiotic treatments. For example, in the current study population at a threshold of 2%, the NeoSeD score provides a net reduction of 10 per 100 patients. This should be interpreted as a net reduction of 10% in the number of unnecessary antibiotic treatments, without having missed any true septic patient. Panel A and B are derived from the same decision curve analysis. More complete guidance regarding interpreting decision-curve analysisis provided in Supplementary data Appendix S1.

**Figure 9:**
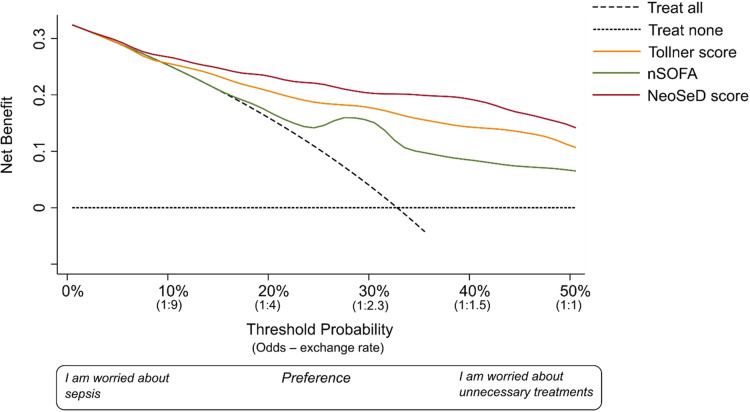
Decision curve analysis for the early detection of blood culture-confirmed sepsis in critically-ill neonates admitted to the neonatal intensive care unit for the newly developed Neonatal Sepsis Diagnostic (NeoSed) score (red line), the Tollner score (orange line), and the neonatal Sequential Organ Failure Assessment (nSOFA, green line). The net benefit (y axis) is a metric representing the benefit of a certain intervention minus its harms, multiplied by an exchange rate. The unit of net benefit is true positives. A net benefit of 0.10, for instance, means “10 true positives for every 100 patients in the target population.” The net benefit is plotted over a range of possible decision thresholds/exchange rates (i.e. individual predicted probabilities given in Figure 3) for the early diagnosis of sepsis (x-axis). The net benefit also incorporates any consequence (i.e., clinical actions) of a diagnosis of sepsis. The net benefit of five different strategies is compared. The two extreme−default−strategies are “treat all” (diagonal dashed line) and “treat none” (orizontal dashed line) meaning enacting clinical actions as if all patients with suspected sepsis were septic (i.e., “treat all”), or as if they were not (i.e., “treat none”). The x-axis can be also renamed as preference: clinicians more worried about the harms of a missed diagnosis of sepsis will adopt thresholds closer to a predicted probability of zero (i.e., left side of the graph), while clinicians more worried about the harms of unnecessary interventions (i.e., on false positives) will adopt higher thresholds (i.e., right side of the graph). The x-axis is also called “exchange rate” which is an odds and represents how many false positives are worth one true positive (i.e., adopting a threshold probability of 10% means that a patient with a predicted probability over 10% will be considered septic—and treated accordingly—and by adopting this classification rule/threshold, one accepts that one true positive is worth nine false positives). Interventions associated with different harms may need the adoption of different thresholds/exchange rates for their applications. The strategy corresponding to the highest net benefit over the largest range of threshold probabilities, should be considered as the best one to adopt. For a more complete guidance regarding interpreting decision-curve analysis, please see Supplementary Appendix S1.

The nomogram plot displaying the predicted probability of sepsis and septic shock according to the NeoSeD score is presented in Figure 7. We examined the relationship between a range of threshold probabilities for predicting sepsis and the relative value of false-positive and false-negative results (i.e., the net benefit) by using decision curve analysis (28) (a guide on how to interpret decision-curve analysis is provided in Supplementary data-Appendix S1). When compared with the strategy of considering as septic all patients with a suspect of sepsis−and treating them accordingly−the ability of the NeoSeD score to predict sepsis provided a better net benefit and a larger net reduction in unnecessary interventions compared to a “treat all” strategy at most threshold probabilities (Figure 8). Importantly, the NeoSeD score was never worse than “treat all” and “treat none” strategies at any threshold probability. The NeoSeD score provided a better net benefit at all decision thresholds for sepsis and blood culture-confirmed sepsis, as compared with the predicted probabilities provided by applying the Tollner and the nSOFA scores to our cohort. In conclusion, the NeoSeD score provided the largest overall net benefit among all other strategies plotted in decision-curve analyses and the largest net reduction in unnecessary interventions (Figures 8, 9).

## Discussion

We developed and internally validated a multivariable, practical diagnostic score for sepsis in ill neonates showing remarkable performances in the development cohort. The NeoSeD score showed a larger net benefit and better discrimination capacity in detecting neonatal sepsis and septic shock than the Tollner and nSOFA scores in our population of critically-ill patients admitted to NICU with suspected sepsis. A10, GA, CRP, the presence of considerable change in skin color, liver enlargement, and neutrofil left shift were identified as the most robust predictors and were included in the NeoSeD score. Importantly, the score retained good calibration and performance measures and offered the best net benefit compared to other available strategies for identification of neonates with confirmed sepsis.

Despite the advances in neonatal care, neonatal sepsis remains a major cause of morbidity and mortality worldwide, reflecting the increased survival rate of extremely preterm neonates with prolonged hospitalization, and the growing antibiotic resistance of bacteria (33, 34).

Sepsis in neonatal population, and especially in very preterm neonates, is not only a life-threatening situation but also a potential precursor for longterm morbidity and neurodevelopmental deficiencies. Various short-term and long-term complications of sepsis in the neonatal population have been recorded. Especially preterm neonates are at increased risk of severe adverse outcomes due to immaturity of their immune mechanisms and multiple invasive interventions. GA and birth weight are inversely and significantly correlated with the occurrence of sepsis (35–38). This correlation was corroborated by our results, as GA was found to be one of the predicting variables for sepsis in our model. Concomitant conditions, which are common in preterm neonates, including patent ductus arteriosus, respiratory distress syndrome, and necrotizing enterocolitis, may also increase the risk of neonatal sepsis. This is mainly due to more invasive therapeutic interventions (mechanical ventilation, central or peripheral vascular access, and prolonged parenteral nutrition) required in this patient subpopulation (37, 39).

The skin reflects early changes in circulation and perfusion attributed to systemic septic reaction (40). Alteration in skin color, a prolonged capillary refill time, and temperature instability, all markers of impaired peripheral vascular reactivity, are evident in septic neonates often before traditional indexes of sepsis (41). Skin color changes demonstrated predictive value in our model. Several biomarkers, including complete blood count components and peripheral smear, CRP, procalcitonin, interleukines, cytokines and chemokines, have been investigated for diagnosis and severity assessment of septic neonates (42). A parameter used in neonatal sepsis evaluation is the neutrophil left shift, a rapid and simple test with considerable positive predictive value and specificity (43, 44). CRP is a useful biomarker with a sensitivity ranging between 9% and 83%, higher specificity (>90%), and negative predictive value in serial measurements. In addition, monitoring CRP levels has been used to evaluate response to treatment (45, 46). Its combined use with other biomarkers has been suggested (46). Left shift of neutrophils and CRP levels emerged as predicting variables in our model. Multi-organ dysfunction often complicates the course of neonatal sepsis. In this context, palpable liver may be a sign of cardiac dysfunction or cardiogenic shock (4). Liver enlargement, observed in neonatal sepsis, also presented as a predictive variable in our model.

Severe sepsis is often complicated by systemic activation of coagulation cascade (4). Hemostasis and inflammation are significantly correlated. Activation of coagulation mechanism and impairment of anticoagulant pathways are both triggered by pro-inflammatory cells, cytokines and chemokines. Endothelial plasminogen activator inhibitor 1 (PAI-1) is induced by cytokine production, leading to inactivation of fibrinolytic system and a subsequent fibrin microvascular deposition. This is the fundamental pathophysiological process of DIC, which results in organ dysfunction. Consumption of coagulation factors and platelets, as well as reduced platelet functionality in septic patients is associated with increased risk of bleeding (4). Prompt diagnosis and management of hemostatic disorders in neonates with sepsis is essential for optimizing care of these patients (47). Conventional coagulation tests, including prothrombin time (PT) and activated partial thromboplastin time (aPTT), and platelet count, present limitations in the evaluation of the global hemostatic profile (48). However, viscoelastic tests assess the overall dynamics of clot formation and lysis, evaluating all stages of the coagulation process (48). Among the ROTEM parameters, EXTEM A10 emerged as the most potent variable in our model for sepsis diagnosis in neonates. This is a parameter which encloses platelet count, platelet function, fibrinogen concentration, factor XIII contribution in clot amplitude, and fibrin polymerization (49). This finding is in line with existing evidence of a hypocoagulable profile, reflected by reduced EXTEM A10, in septic neonates with hemorrhagic diathesis (11). Furthermore, EXTEM A10 has been independently and significantly associated with increased risk of mortality in critically ill neonates (50), while incorporating this variable in selected well- established neonatal disease severity scoring systems may improve their predictive performance for mortality (51). Finally, EXTEM A10 is included as a variable in the NeoBRis score for prediction of 24-hour bleeding risk in critically ill neonates (23, 52).

Sepsis complications could be devastating and life-threatening in neonates. A positive blood culture is the gold standard for diagnosis of sepsis. However, this is not feasible until 24–48 h following clinical presentation (53), while blood culture-negative cases account for a significant percentage of septic neonates (54). In addition, initial signs and symptoms of sepsis are subtle and often non-specific in neonates, and there is no established uniform definition for sepsis in this population (15). Thus, treatment with antibiotics is usually initiated early in the course of presumed sepsis, and as a consequence, a significant proportion of neonates receives antibiotics unnecessarily (15, 33). This practice is largely responsible for a high rate of antibiotic resistance in NICUs, which ultimately further aggravates the outcome for septic neonates. It has been estimated that 31% of neonatal deaths related to sepsis can be attributed to antimicrobial resistance, rendering it a major issue for global healthcare systems (55). Antimicrobial stewardship programs are necessary for avoidance of antibiotics overuse, while prompt management of suspected sepsis is also paramount (56). In this direction, several attempts have been made over the last few years to develop sepsis scores in neonatal population, either for EOS or for LOS (57–61). A free, online neonatal EOS-risk calculator (https://neonatalsepsiscalculator.kaiserpermanente.org) was developed for prediction of EOS incidence and for guiding decisions on treatment with antibiotics (62). Widespread application of this calculator has reduced antibiotics administration without a compromise in safety, especially in populations with relatively low rate of culture-proven EOS and uninhibited access to follow-up care (46). However, as half of the patients with culture-proven EOS are asymptomatic at birth, close monitoring and clinical observation is required to ensure safety. Critical limitations of this scoring system include the implementation only for EOS and in neonates ≥34 weeks GA. In 1982, Töllner et al. (21), developed a score system based on 7 clinical parameters (skin color, capillary refill time, muscular hypotonia, apnea, respiratory distress, hepatomegaly, gastrointestinal symptoms) and 3 hematologic parameters (WBC count, neutrophil left shift and thrombocytopenia). The discrimination capacity of the NeoSeD score was significantly higher compared to the Töllner score in our study population for both sepsis (*p* < 0.001) and septic shock (*p* = 0.043). This superiority could be due to a more comprehensive evaluation of coagulation status and the adoption of prematurity as a risk factor for neonatal sepsis. The NeoSeD score was able to predict the entire spectrum of individual probability of sepsis and sepsis shock, and showed optimal calibration of probabilities from the lowest to the highest-risk neonates. Although we performed extensive internal validation, we must recognize that without an external validation, model performance may be optimistic (63). In adults with suspected infection, SOFA score is used to estimate risk of Intensive Care Unit admission or mortality. The nSOFA, an adaptation of SOFA for neonates, was developed as an assessment tool for prediction of mortality risk of LOS in very low birth weight infants (64). Further to its notable performance, nSOFA was suggested to help build a consensus definition of sepsis in this population (22). In the present study, the discrimination capacity of NeoSeD score was significantly higher than that of nSOFA for both sepsis and septic shock. This could possibly be attributed to the fact that nSOFA score was designed for prediction of sepsis outcome, while NeoSeD score was specifically developed for the diagnosis of sepsis.

Although NeoSeD score was primarily designed to identify neonates with sepsis, it also demonstrated excellent performance for the detection of septic shock. As the presentation of sepsis is non-specific in neonates, advancement to septic shock is a possible, early complication, with an adverse impact on prognosis. Consequently, time is of the utmost essence for the reversion of neonatal shock.

Prompt evaluation and identification of sepsis symptoms and signs is crucial for the early and appropriate management of the neonate. To date, there is no single, optimal biomarker to diagnose sepsis, therefore, treatment decisions should be based on a combined approach taking into consideration both risk factors and neonatal clinical status. Among our critically ill neonates evaluated for suspected sepsis, the prevalence of confirmed sepsis was 41%. This finding is in agreement with previously reported rates, highlighting a high prevalence of non-septic neonates who receive antibiotics (65, 66). A scoring system with optimal performance for diagnosis of sepsis in ill neonates assessed for sepsis would be ideal. In the process of our model development, the most frequently used in clinical practice and existing scoring systems risk factors, demographic and laboratory data, and clinical signs, were assessed. Sepsis diagnostic scores based on clinical symptoms and signs are easily accessible and extremely helpful in limited-resource settings, while scores including primarily laboratory values provide a more accurate and objective tool. However, laboratory assessment is time- consuming and requires respective equipment. Therefore, the incorporation of clinical and readily available laboratory variables in a scoring model may be optimal (67).

Finally, the NeoSeD score allows for neonatal sepsis and septic shock risk stratification, providing clinicians with a more detailed therapeutic guidance. We demonstrated the clinical utility of the NeoSeD score in our study population by applying decision curve analysis (28), an approach whose application has been highly recommended recently (68–70). Irrespective of the threshold chosen for a diagnosis of sepsis, the NeoSeD score provided the highest overall net benefit among all strategies plotted in decision curve analyses. The analyses also suggested that even at low threshold probabilities (<4%) the NeoSeD score may offer a benefit in terms of net reduction of unnecessary interventions compared to a “treat all” strategy, which is the default strategy in most NICUs. This confirmatory step justifies the potential clinical applicability of the NeoSeD score.

The relatively small sample size and the single center origin are the main limitations of this study. On the other hand, a unique center study is characterized by homogeneity of methods, practices, and recording of data. External validation through multicenter studies with larger datasets from other NICUs is necessary to evaluate the generalizability of NeoSeD model prior to its clinical application.

In conclusion, the proposed model is simple, accurate, practical, and its application may contribute to the timely diagnosis and appropriate treatment of hospitalized neonates evaluated for sepsis, especially in case of delayed or negative blood culture results. By incorporating signs and symptoms, laboratory results, and risk factors, NeoSeD score could potentially improve early identification, optimal management, and short- and long-term outcome of septic neonates.

## Data Availability

The raw data supporting the conclusions of this article will be made available by the authors, without undue reservation.
