# Supplementary material for: Development and validation of a sepsis diagnostic scoring model for neonates with suspected sepsis
Source: Front Pediatr. 2022 Oct 6;10:1004727. doi: 10.3389/fped.2022.1004727 (PMC9582514; doi:10.3389/fped.2022.1004727)
Supplement: Supplementary file 1 [file Table1.docx]

**Supplementary Table 1.** Characteristics of the study population (N = 291).

|  | **Mean ± SD; median (IQR) or n (%)** |
| --- | --- |
| Gender (males; N = 290) | 181 (62.4) |
| Gestational age (weeks; N = 290) | 34.7 ± 4.65; 37 (30–38) |
| Birth weight (g; N = 290) | 2341 ± 1051; 2505 (1260–3240) |
| Cesarean section (N = 290) | 203 (70.0) |
| 5-minutes Apgar score (N = 290) | 8.24 ± 1.40; 8 (8–9) |
| Body temperature (N = 290) | 37.0 ± 0.50; 37 (36.7–37.2) |
| Mean blood pressure (N = 289) | 52.1 ± 8.92; 52 (46–58) |
| Change in skin color (N = 290) |  |
| Normal | 31 (10.7) |
| Moderate change | 182 (62.8) |
| Considerable change | 77 (26.5) |
| capillary refill time (CRT) (N = 290) |  |
| Normal | 80 (27.6) |
| Impaired | 149 (51.4) |
| Considerably impaired | 61 (21.0) |
| Base deficit (mmol/L; N =290) | 3.56 ± 2.99; 3 (1.2–5) |
| Blood pH (N = 290) |  |
| ≥ 7.30 | 259 (89.3) |
| ≥ 7.20 and < 7.30 | 27 (9.3) |
| < 7.20 | 4 (1.4) |
| Shift to the left (N =288) |  |
| No | 192 (66.7) |
| Moderate | 85 (29.5) |
| Considerable | 11 (3.8) |
| Muscular hypotonia (N = 286) | 138 (48.2) |
| Bradycardia (N =290) | 8 (2.8) |
| Apneic spell (N = 287) | 81 (28.2) |
| Liver enlargement (N =290) | 55 (19.0) |
| Gastrointestinal symptoms (N = 289) | 121 (41.9) |
| Respiratory distress (N =290)* | 95 (32.8) |
| SpO_2_/FiO_2_ (N = 290) |  |
| Not intubated or intubated with SpO_2_/FiO_2_ ≥300 | 149 (51.4) |
| Intubated with 200≥ SpO_2_/FiO_2_ <300 | 100 (34.5) |
| Intubated with 150≥ SpO_2_/FiO_2_ <200 | 22 (7.6) |
| Intubated with 100≥ SpO_2_/FiO_2_ < 150 | 12 (4.1) |
| Intubated with SpO_2_/FiO_2_ <100 | 7 (2.4) |
| Tollner score (N =291) | 7.15 ± 4.40; 6 (4–10) |
| nSOFA (N = 291) | 2.84 ± 3.00; 2 (0–4) |
| NeoBAT score (N = 290) | 0.46 ± 0.91; 2 (0–4) |
| Seizures (N = 290) | 13 (4.5) |
| Diuresis (ml/kg/h; N = 290) |  |
| ≥ 1 | 281 (96.9) |
| ≥ 0.1 and <1 | 7 (2.4) |
| < 0.1 | 2 (0.7) |
| Edema (N = 288) |  |
| No | 243 (84.4) |
| Subcutaneous | 41 (14.2) |
| Generalized | 4 (1.4) |
| Respiratory distress syndrome (N =290)* | 177 (61.0) |
| Bronchopulmonary dysplasia (N = 288) |  |
| No | 215 (74.1) |
| Mild | 20 (6.9) |
| Moderate | 11 (3.8) |
| Severe | 44 (15.2) |
| Retinopathy of prematurity (N =290) |  |
| No | 224 (77.3) |
| Yes | 43 (14.8) |
| Yes, requiring laser treatment | 23 (7.9) |
| Patent ductus arteriosus (N = 288) |  |
| No | 259 (89.9) |
| Yes, requiring no treatment | 5 (1.8) |
| Yes, requiring medical treatment | 22 (7.6) |
| Yes, requiring surgical treatment | 2 (0.7) |
| Hypotension (N =290) |  |
| No | 217 (74.8) |
| Pharmacological support to maintain adequate BP | 53 (18.3) |
| Hypotension despite pharmacological support | 20 (6.9) |
|  |  |
| Multiple organ distress syndrome (N = 290) | 65 (22.4) |
| Intra-ventricular hemorrhage (N = 290) | 93 (32.1) |
| Gastrointestinal hemorrhage (N = 290) | 26 (9.0) |
| Pulmonary hemorrhage (N = 290) | 12 (4.1) |
| Venipuncture site hemorrhage (N = 290) | 57 (19.7) |
| Urinary tract hemorrhage (N = 287) | 8 (2.8) |
| Periventricular leukomalacia (N = 290) | 37 (12.8) |
| Intrauterine growth retardation (N = 290) | 38 (13.1) |
| Acute renal failure (N = 290) | 37 (12.8) |
| Disseminated intravascular coagulopathy (N = 290) | 45 (15.5) |
| Necrotizing enterocolitis (N = 289) | 9 (3.1) |
| Albumin (g/dL; N = 287) | 2.51 ± 0.43; 2.5 (2.2–2.7) |
| Hematocrit (%; N = 275) | 40.1 ± 6.52; 39.5 (35.9–44.1) |
| WBC (×10^3^, cells/µL; N = 290) | 14.6 ± 8.59; 12.7 (9.44–18.3) |
| Neutrophils (%; N = 289) | 58.5 ± 18.1; 60.5 (47.0–72.6) |
| Platelets (×10^3^, cells/µL; N = 290) | 215 ± 142; 209 (89.0–296) |
| C-reactive protein (mg/L; N = 290) | 37.2 ± 43.5; 18.5 (4.4–54.4) |
| SGOT (IU/L; N = 290) | 84.5 ± 167; 50 (31–80) |
| SGPT (IU/L; N = 289) | 36.7 ± 76.4; 18 (12–34) |
| Total bilirubin (mg/dL; N = 290) | 7.26 ± 6.64; 6.0 (3.1–9.6) |
| Direct bilirubin (mg/dL; N = 287) | 1.25 ± 4.20; 0.3 (0.2–0.5) |
| Blood urea nitrogen (mg/dL; N = 290) | 39.2 ± 34.9; 28 (19–50) |
| Creatinine, mg/dL (N = 290) | 0.61 ± 0.36; 0.5 (0.4–0.8) |
| *Extem ROTEMS parameters:* |  |
| CT (minutes; N = 291) | 62.5 ± 71.0; 51.0 (44.0–61.0) |
| A5 (mm; N = 291) | 42.1 ± 14.4; 43.0 (33.0–53.0) |
| A10 (mm; N = 290) | 51.2 ± 15.0; 53.0 (43.0–63.0) |
| A15 (mm; N = 288) | 54.7 ± 14.7; 57.0 (47.0–66.0) |
| A20 (mm; N = 289) | 56.5 ± 14.3; 59.0 (49.0–67.0) |
| A25 (mm; N = 289) | 57.2 ± 14.1; 60.0 (51.0–68.0) |
| A30 (mm; N = 279) | 57.6 ± 14.1; 60.0 (51.0–68.0) |
| CFT (minutes; N = 288) | 157± 316; 82.5 (59.0–127) |
| MCF (mm; N = 291) | 58.8 ± 14.8; 60.0 (51.0–69.0) |
| ALPHA (°; N = 290) | 72.8 ± 12.2; 76.0 (70.0–80.0) |
| LI30 (%; N = 275) | 98.8 ± 6.02; 100 (99.0–100) |
| LI45 (%; N = 260) | 96.7 ± 5.95; 98.0 (96.0–100) |
| LI60 (%; N = 236) | 94.2 ± 6.28; 95.0 (92.0–98.0) |
| MCE (N = 270) | 171 ± 114; 151 (104–214) |
| ML (%; N = 290) | 9.56 ± 11.8; 8 (3–12) |

*Abbreviations:* BP, blood pressure; SD, standard deviation; IQR, interquartile range; CT, clotting time; CFT, clot formation time; A5, clot amplitude at 5 minutes; A10, clot amplitude at 10 minutes; A15, clot amplitude at 15 minutes; A20, clot amplitude at 20 minutes; A25, clot amplitude at 25 minutes; A30, clot amplitude at 30 minutes; MCF, maximum clot firmness; LI60, lysis index at 60 min; ML, maximal lysis; MCE, maximum clot elasticity; SGOT, serum glutamic oxaloacetic transaminase; SGPT, serum glutamic pyruvic transaminase, WBC, white blood cells, NeoBAT, neonatal bleeding assessment tool; nSOFA, neonatal, Sequential Organ Failure Assessment

Footnotes: *Respiratory Distress Syndrome (RDS) was a diagnosis throughout hospitalization; respiratory distress refers to symptoms occurring at the time of evaluation due to suspected sepsis.
