# Supplementary material for: Development and validation of a sepsis diagnostic scoring model for neonates with suspected sepsis
Source: Front Pediatr. 2022 Oct 6;10:1004727. doi: 10.3389/fped.2022.1004727 (PMC9582514; doi:10.3389/fped.2022.1004727)
Supplement: Supplementary file 2 [file DataSheet1.docx]

**Supplementary data**

**1. METHODS**

**1.1 Inclusion criteria**

The presence of the following clinical signs and/or laboratory data suggestive of sepsis necessitating initiation of antibiotic therapy ([1](#_ENREF_1), [2](#_ENREF_2)) :

Clinical signs: (i) temperature instability (core temperature of >38.0°C or <36°C or rectal-skin temperature difference of more than 3.5 degrees C) and poor feeding; (ii) abdominal distension, hepatomegaly; (iii) apnea, dyspnea, retractions, need for oxygen or ventilation support; (iv) bradycardia/tachycardia (bradycardia defined as a mean heart rate <10th percentile for age in the absence of β-blocker drugs or congenital heart disease; or otherwise unexplained persistent bradycardia or tachycardia, defined as a mean heart rate>2 standard deviations (SD) above normal for age in the absence of external stimulus, chronic drugs, or painful stimuli; or otherwise unexplained persistent elevation over a 0.5- to 4-hr time period), poor peripheral perfusion, hypotension (decrease in blood pressure <5th percentile for age or systolic blood pressure >2 SD below normal for age or need for vasoactive drug to maintain blood pressure in normal range); (v) irritability, lethargy and/or hypotonia; (vi) jaundice, pallor, petechiae. Risk factors for infection from perinatal history include premature rupture of membranes (>18 hours), maternal fever during labor, intra-amniotic infection, and chorioamnionitis.

Laboratory findings: leukopenia, defined as white blood cells (WBC) <5,000/mm^3^; absolute neutrophil count (ANC) <1,500/mm^3^ or ANC >5,400/mm^3^; Immature to Total Neutrophil (I/T) Ratio >0.2; thrombocytopenia, defined as platelets (PLT) <100,000/mm^3^; C-reactive protein (CRP) levels >10 mg/L; and metabolic acidosis (base excess <−10 mEq/L).

Complete blood counts were performed on Sysmex XE-2100 analyzer (Roche, IL, USA). Peripheral blood smears prepared with Giemsa stain were examined, and band forms, myelocytes and metamyelocytes in leukocyte formula evaluated as immature neutrophils and I/T ratio were calculated. Blood specimens were obtained from all study neonates using a sterile technique and were inoculated into an aerobic blood bottle (BacT Alert PF, Biomerieux, LYON, France). The organisms were identified based on gram staining and growth on agar media.

All biochemical parameters were measured by EXL DIMENSION Analyzer (SIEMENS, Healthcare Diagnostics, Newark, DE, USA). CRP method was based on a particle enhanced turbidimetric immunoassay technique (PETIA).

The following conditions were recorded for all study neonates:

Respiratory Distress Syndrome (RDS): The diagnosis of RDS was based on the clinical status of a preterm neonate with increasing oxygen requirements shortly after birth, along with characteristic chest radiograph features([3](#_ENREF_3)).

Bronchopulmonary Dysplasia (BPD): BPD, a chronic neonatal lung disease of preterm neonates, is defined as need for supplemental oxygen for at least 28 days. BPD is classified as mild, moderate, or severe according to the concentration of oxygen required (<30% vs. ≥30%) and the mode of respiratory support necessary at 36 weeks’ postmenstrual age ([4](#_ENREF_4)).

Disseminated Intravascular Coagulopathy (DIC): For the diagnosis of DIC, the neonatal diagnostic criteria of the Japan Society of Obstetrical, Gynecological & Neonatal Hematology ( JSOGNH) were used ([5](#_ENREF_5)).

Acute renal failure or Acute Kidney Injury (AKI): Neonatal AKI was defined based on the relevant diagnostic criteria ([6](#_ENREF_6)).

**1.2 Statistical analysis and model development**

We presented the baseline characteristics and ROTEM parameters of the study population as means ± standard deviations (SDs), medians and interquartile ranges (IQRs), or percentages, when appropriate.

Our aim was to build a practical, multivariable diagnostic score for the prompt diagnosis of sepsis in neonates, using ROTEM parameters and clinical and biochemical characteristics.

Initially, we investigated the association between ROTEM parameters and sepsis. We assessed multicollinearity by using the non-parametric Spearman correlation test. Most parameters were very highly correlated (rS > 0.80 and p < 0.05). To avoid substantial multicollinearity in our model development, the ROTEM parameter most associated with sepsis in a univariable logistic regression (ie, highest likelihood ratio chi-squared test), ie, A10 was retained as the candidate predictor ([7](#_ENREF_7)). Other candidate predictors for the multivariable model development included gender, GA, birthweight, delivery mode, and potential maternal risk factors for sepsis (prolonged and/or premature rupture of membranes, fever, chorioamnionitis, positive colonization with GBS οr history of previous infant with GBS infection and GBS bacteriuria). The evaluated clinical variables included 5-minutes Apgar score, temperature instability, jaundice, cyanosis, edema or sclerema, petechiae, poor peripheral perfusion assed by capillary refill time, change in skin color, poor feeding, irritability, seizure, lethargy, gastrointestinal symptoms, vomiting, abdominal distention, hepatomegaly, apnea, tachypnea, need for respiratory support, grunting, tachycardia, bradycardia, mean blood pressure, hypotension, and urine output. Possible predictors within laboratory data included blood pH, base deficit, plasma lactate levels, PaO2/FIO2, hematocrit level, white blood cells, neutrophil count and neutrophil left shift, platelet count, CRP, serum albumin, SGOT, SGPT, total bilirubin, direct/indirect bilirubin, and plasma creatinine. The distribution of each candidate continuous variable was inspected. Prior to any analysis, we applied a log10-transformation to serum albumin, white blood cells, CRP, SGOT, SGPT, total bilirubin, direct bilirubin, indirect bilirubin, creatinine, as their distribution was severely right skewed.

The multivariable model was selected and fitted my means of L1-penalized least absolute shrinkage and selection operator (LASSO) logit regression ([8-10](#_ENREF_8)). The LASSO is especially useful in case of high-dimensional datasets (ie, when the ratio of the number of events per candidate predictors is low to very low) as it is is less prone to multicollinearity, misclassification, overfitting, and optimism in the predictive performance ([8-11](#_ENREF_8)). As this procedure is sensitive to the absolute size of the coefficients, we first standardized the variables. The LASSO penalizes the absolute size of the coefficients of a multivariable regression based on the value of a penalty parameter λ. ([9](#_ENREF_9), [12](#_ENREF_12)). With larger penalties, the coefficients of weaker candidate predictors shrink to zero, and only the best predictors are retained in the model. The value of λ was selected by using a 10-fold cross validation process ([12](#_ENREF_12)). We used the value of λ corresponding to the most parsimonious model within one standard error of the minimum of the cross-validation function, as recommended ([13](#_ENREF_13)). Model fitness was checked by using the Hosmer-Lemeshow test which is a goodness-of-fit test comparing observed and expected probabilities into quantiles of linear predictor ([14](#_ENREF_14)). Missing data did not exceed 2% for any candidate predictor. We considered them as missing at random and we performed a complete-case analysis.

We developed a diagnostic score [Neonatal Sepsis Diagnostic (NeoSeD) score] by converting the β coefficient of each predictor in the final model into a weighted score using integer values while preserving monotonicity and simplicity. We reported the full model specifications in a table including the model intercept, as recommended by the TRIPOD guidelines ([11](#_ENREF_11)). We produced a nomogram plot transforming all possible total point scores into the corresponding absolute risk of sepsis. Finally, we performed a decision curve analysis as recommended by Vickers et al.([15](#_ENREF_15)) to assess the clinical utility of the NeoSeD score by quantifying the net benefit when different threshold probabilities for a diagnosis of sepsis were considered (a guide to interpreting decision curve analysis is provided in the Supplementary Appendix 1).

**1.3 Internal validity, calibration and performance measures**

We selected variables entering the final model through 10-fold cross-validation (CV) LASSO logit regression, a procedure maximizing the internal validity (ie, reproducibility) of the resulting model ([11](#_ENREF_11)). In k-fold CV the dataset is equally splitted in 10 parts. The first fold is treated as a validation set, and the regression is fit on the remaining 9 folds and a test error is calculated. This procedure is repeated 10 times; each time a different fold is treated as the validation set. A CV-function is produced by averaging the test error across the 10 runs. The entire procedure is designed to select the λ parameter corresponding to the minimum test error (or within one standard error of it) ([13](#_ENREF_13)) in the CV-function as to minimize the out-of sample error and avoid fitting a model with excessive optimism ([12](#_ENREF_12)).

We verified the calibration of our final model through a predicted versus observed probability plot: the calibration belt ([16](#_ENREF_16)). In a perfectly calibrated model the probabilities estimated by the model accurately match the observed outcome across the spectrum of predicted probabilities. In such case, the calibration belt should lie on the 45° line (ie. bisector) in the resulting graph and its 95% CI should not be under or over the bisector at any point. A calibration belt below the bisector would mean that, within that interval of risk, the statistical model overestimates the observed rate of the event, while the opposite would be true in the alternative case. The calibration belt outperforms other calibration approaches such as locally weighted smoothers and plots of predicted versus observed events across deciles ([17](#_ENREF_17)). To check the presence of any difference between the mean predicted probability and the observed proportion of sepsis cases, we also computed the calibration in the large measure (ie, it should ideally equal zero) and accompanied it with a formal statistical test under the null hypothesis of no difference ([11](#_ENREF_11)).

The discrimination capacity of our preliminary diagnostic score was assessed through receiver operating characteristic (ROC) analyses which allowed the calculation of the area under curve (AUC) ([11](#_ENREF_11)). Given that external validation was not feasible in the current study, we tested the ability of the model to generalize to new cases by using 10-fold cross validation. We calculated the discrimination capacity of our model in each of the 10-fold cross validation subsamples and averaged it reporting the mean cross-validated AUC to provide a more realistic measure of model performance. We also calculated the brier score as a measure of diagnostic accuracy ([18](#_ENREF_18)). The brier score ranges from 0 (perfect accuracy, ie, all diagnoses are accurate) to 1 (ie, all diagnoses are wrong) with a value of 0.25 meaning a diagnostic accuracy equal to tossing a coin. The proportion of variance explained by the model on the logit scale was calculated with the McKelvey–Zavoina pseudo R2, taking values from 0 (ie, no variance explained) to 1 (ie, the entire variance is explained) ([19](#_ENREF_19)).

The net benefit and the area under curve of the NeoSeD score were formally compared with those of the Tollner and those of the nSOFA scores ([20-22](#_ENREF_20)). Though the NeoSeD score was developed as a diagnostic tool for sepsis, we also tested its overall performance on septic shock (secondary outcome measure) and on blood culture-confirmed sepsis (sensitivity analysis). All tests were two-sided. The STATA (Stata Corp., College Station, Texas, United States) and R software were used for statistical modeling and analysis. A two-sided p-value <0.05 was considered statistically significant.

**2. SUPPLEMENTARY APPENDIX 1**

**2.1 Interpreting decision curve analysis: a guide for readers**

**2.1.1 *What is a decision curve analysis?***

A decision curve analysis is a method to assess the clinical utility of one or more prognostic, diagnostic score/rule or even biological marker. It was introduced for the first time by Vickers and colleagues in 2006 ([15](#_ENREF_15)). Performing decision curve-analysis has been recently highly recommended by editorials in many top journals including JAMA, BMJ, and Journal of Clinical Oncology ([23-25](#_ENREF_23)). A decision curve analysis is typically represented in a graph plotting the net benefit of certain prognostic or diagnostic scores/rules against a range of plausible threshold probabilities to reach a clinical decision.

***2.1.2 Why does it exist? What does it add?***

Sensitivity, specificity, discrimination (i.e. AUC or c-statistics) and other measures of performance and calibration, are not able to give a comprehensive and interpretable information about the clinical value of a certain prognostic or diagnostic score/rule, especially comparing to other existing treatment strategies.

A decision curve-analysis measures the net benefit (i.e. the relative value of false-positive and false-negative results), which inherently incorporates the consequences of the decisions taken (including any treatment, but also change in monitoring, or even advice in lifestyle change) on the basis of a certain score or rule.

*Example:*

In the current study, the decision curve for the NeoSeD score (**Fig. 4 panel A**) displays the net benefit of deciding which patient is septic and which one is not over a range of possible thresholds for this decision (i.e. x axis, also named as “preference”). The net benefit displayed incorporates inherently the consequences of a certain diagnosis such as giving intravenous broad-spectrum antibioticsThe net benefit is plotted over a range of predicted probabilities (more on this on the next sections).

***2.1.3 How is the net benefit calculated?***

The net benefit is defined as *benefit−(harm×exchange rate)* ([23](#_ENREF_23)). We will see more on the exchange rate in the next section. This can be also written as *sensitivity × prevalence–(1–specificity) × (1–prevalence) × exchange rate* where the exchange rate is the odds at the threshold probability (i.e. applied to the current study, if individual p_predicted_>p_threshold_ then a patient is considered with sepsis) ([26](#_ENREF_26)).

***2.1.4 How to interpret the net benefit?***

In respect to other measures of performance or calibration, the net benefit on the y axis has a straightforward and practical interpretation. The unit of net benefit is true positives. A net benefit of 0.10, for instance, means “10 true positives for every 100 patients in the target population.”

*Example:*

As illustrated in the footnotes of **Fig. 4 panel A**, the net benefit of the NeoSeD score of – roughly – 0.35 at a threshold probability of 0.10 can be interpreted as:

“Comparing to treat nobody while waiting for the results of the blood culture, taking clinical decisions on the basis of the NeoSeD score at this threshold probability is the equivalent of a strategy that found 35 sepsis per 100 patients with suspected sepsis – and treated them accordingly – without having given any unnecessary treatment”.

***2.1.5 What is the meaning of the x axis (preference)?***

The x axis is the range of probability thresholds (i.e. predicted by the model, in our case the probability given in the nomogram in **Fig. 3**) upon which a clinician can choose to decide whether the patient has the outcome or not and base his/her clinical decisions accordingly. It is also called exchange rate, or the number of “false positives” that are worth one true positive. The x axis displays a spectrum of plausible thresholds/exchange rates and is often renamed as “preference”.

***2.1.6 How to interpret a decision curve analysis***

Using as explanatory example this study, a clinician who is more worried about the consequences of a diagnosis of sepsis and septic shock (e.g. mortality) then of the harm of the intervention will choose a low or very low threshold (i.e. the left side of the graph with thresholds close to zero) to take a certain clinical decision. In other words, the clinician will likely accept to treat a high or very high number of false positives to treat effectively one true positive (e.g. 1:100 or even 1:1000 corresponding roughly to threshold probabilities of 0.01 and 0.001, respectively). This scenario is plausible for interventions that have low risk profiles in comparison with the risk of sepsis.

On the contrary, a clinician more worried about the possible harms of the intervention than about the harms of sepsis will prefer higher thresholds (i.e. the middle or right side of the graph). In other words, the clinician will likely accept to treat a low or moderate number of false positives to treat effectively one true positive (e.g. 1:4 or 1:9 corresponding to threshold probabilities of 0.20 and 0.10, respectively). This scenario is plausible for all interventions that have moderate or high-risk profiles.

***2.1.7 What is the relevance of a decision curve analysis?***

More invasive/risky interventions will have plausibly different (i.e. higher) thresholds/exchange rate for the same clinician.

For this reason, it would be ideal to demonstrate that a new score is never worse (i.e. does never provide a lower net benefit) than the two extreme strategies of “treat all” (the diagonal dashed line in **Fig. 4 panel A**) or “treat none” (the horizontal dashed line in **Fig. 4 panel A**) over a range of plausible exchange rates/thresholds considering a spectrum of possible interventions and preferences. Preferences mean clinicians’ preferences, but also, patients’ preferences.

When other management strategies are available, the new score should be an improvement over these existing strategies and should show larger net benefits at least in certain ranges of exchange rates (x axis), and, ideally, over the entire range of plausible thresholds. This is the case for the NeoSeD score when compared to the Tollner and nSOFA scores and when compared to the two extreme strategies of “treating all” and “treating none”.

***2.1.8 Net benefit can also be expressed as number of interventions avoided***

In cases in which a strategy to treat all is the most commonly adopted strategy (i.e. broad spectrum antibiotics for all neonates with suspected sepsis), net benefit can be conveniently expressed as “net number of interventions avoided” ([26](#_ENREF_26)). This is reported in **Fig.4 panel B.** In this case the y axis expresses the net benefit in terms of “net reduction in interventions per 100 patients” in respect to a strategy to treat all (i.e. the diagonal dashed line in **Fig. 4 panel A**).

For example, by observing **Fig. 3 panel B** we can conclude that following the NeoSeD score and adopting a threshold of 2% to decide whether or not administer broad spectrum antibiotics would yield a net reduction of 10% in antibiotics used, without having missed any true septic patient. Adopting instead a threshold of 15% for another, riskier, intervention X would yield to a net reduction of this intervention of about 20% (**Fig.3 panel B**).

In the current study, irrespectively of the way the net benefit was expressed (panel A *vs.* panel B), the hierarchy of models was the same: the NeoSeD score offered the larger net benefit and the larger net reduction in unnecessary interventions over the spectrum of plausible thresholds for decision making.

**2.2 Frequently asked questions**

**2.2.1 Does a decision curve analysis tell you what is the best threshold to adopt?**

No, it does not. As a general statement, less risky treatments should have a lower threshold probability and more risky treatments should have a higher threshold probability. A decision curve analysis plots the net benefit associated with interventions over a range of plausible thresholds for deciding to use these interventions, but cannot inform about the best thresholds to be used which cannot be determined using purely statistical criteria.

Clinicians should work out a reasonable range of threshold probabilities based on balance of the harms of avoiding a certain intervention for a true positive and unnecessarily intervening on a false positive (i.e. the x axis or exchange rate) ([26](#_ENREF_26)).

**2.2.2 Should there be confidence intervals in decision curves? How much of a difference between curves is enough?**

Although methods to derive confidence intervals for decision curves are now available, statistical significance has little relevance in decision theory and most decision curves are not published with confidence bands ([26](#_ENREF_26)).

However, if performing a certain diagnostic test is associated with significant risks (i.e. morbidity and mortality), discomfort/pain (i.e. certain biopsies), or if using a certain prognostic score is burdensome as dozens of variables that are not routinely collected are needed; then we would likely not use the test/score if it provided only a very small improvement in net benefit over other more simple strategies such as treating all. In these cases, an amount of harm can be incorporated in the decision curve analysis and considered when gauging the expected net benefit over other more convenient/less potentially harmful strategies. Given the ease of use and absence of any significant harm in obtaining the variables to calculate the NeoSeD score, this is not the case in the current study in which a classic decision curve is presented where harms of applying all tests/scores are not formally considered.

**2.2.3 Why do we need decision curves? How can a model with lower AUC be better than another model?**

This is because both discrimination and calibration are taken into account at the same time by decision-curve analysis ([15](#_ENREF_15), [26](#_ENREF_26)). A poorly calibrated model can have exactly the same AUC as another model, but can provide very distorted predicted probabilities, thus predicting an individual probability of event that is even 10 times higher/lower than the real one in respect to the expected. Similarly, two perfectly calibrated models can have very different AUCs.

**2.2.4 Can a decision curve analysis substitute a full decision analysis or cost-effectiveness analysis?**

No, it cannot. Decision curve-analyses are the result of certain assumptions but are much simpler and quicker to perform than a formal, comprehensive, decision analysis involving multiple outcomes, preferences and costs. However, if the results of a decision curve analysis are clear in saying that a certain score has no value over the entire range of plausible threshold probabilities, then a formal decision analysis can be avoided ([15](#_ENREF_15), [26](#_ENREF_26))

**Note:** For an even more comprehensive source of information please see [www.decisioncurveanalysis.org](http://www.decisioncurveanalysis.org) and other published papers on the topic ([15](#_ENREF_15), [23-26](#_ENREF_23)).

**3. REFERENCES**

1. Levy MM, Fink MP, Marshall JC, Abraham E, Angus D, Cook D, et al. 2001 SCCM/ESICM/ACCP/ATS/SIS International Sepsis Definitions Conference. Critical care medicine. 2003;31(4):1250-6.

2. McGovern M, Giannoni E, Kuester H, Turner MA, van den Hoogen A, Bliss JM, et al. Challenges in developing a consensus definition of neonatal sepsis. Pediatric research. 2020;88(1):14-26.

3. Martin R. Pathophysiology, clinical manifestations, and diagnosis of respiratory distress syndrome in the newborn. In: Garcia-Prats JA, editor. UpToDate 2020. UpToDate. Waltham, MA, (Accessed on November 30, 2020.)2020.

4. Jobe AH, Bancalari E. Bronchopulmonary dysplasia. American journal of respiratory and critical care medicine. 2001;163(7):1723-9.

5. Go H, Ohto H, Nollet KE, Kashiwabara N, Ogasawara K, Chishiki M, et al. Risk factors and treatments for disseminated intravascular coagulation in neonates. Ital J Pediatr. 2020;46(1):54-.

6. Selewski DT, Charlton JR, Jetton JG, Guillet R, Mhanna MJ, Askenazi DJ, et al. Neonatal Acute Kidney Injury. Pediatrics. 2015;136(2):e463-73.

7. Sokou R, Piovani D, Konstantinidi A, Tsantes AG, Parastatidou S, Lampridou M, et al. A Risk Score for Predicting the Incidence of Hemorrhage in Critically Ill Neonates: Development and Validation Study. Thrombosis and haemostasis. 2021;121(2):131-9.

8. Tibshirani R. Regression Shrinkage and Selection via the Lasso. Journal of the Royal Statistical Society Series B (Methodological). 1996;58(1):267-88.

9. Steyerberg E, Eijkemans M, Habbema J. Application of Shrinkage Techniques in Logistic Regression Analysis: A Case Study. Statistica Neerlandica. 2001;55:76-88.

10. Dormann CF, Elith J, Bacher S, Buchmann C, Carl G, Carré G, et al. Collinearity: a review of methods to deal with it and a simulation study evaluating their performance. Ecography. 2013;36(1):27-46.

11. Moons KG, Altman DG, Reitsma JB, Ioannidis JP, Macaskill P, Steyerberg EW, et al. Transparent Reporting of a multivariable prediction model for Individual Prognosis or Diagnosis (TRIPOD): explanation and elaboration. Annals of internal medicine. 2015;162(1):W1-73.

12. Gareth James, Daniela Witten, Trevor Hastie, Tibshirani R. An Introduction to Statistical Learning: Springer; 2013.

13. Trevor Hastie, Robert Tibshirani, Wainwright M. Statistical Learning with Sparsity

The Lasso and Generalizations. 1st Edition ed: Chapman and Hall/CRC; 2015.

14. David W. Hosmer Jr. SL, Rodney X. Sturdivant. Applied Logistic Regression. Third Edition ed: John Wiley & Sons, Inc; 2013.

15. Vickers AJ, Elkin EB. Decision curve analysis: a novel method for evaluating prediction models. Medical decision making : an international journal of the Society for Medical Decision Making. 2006;26(6):565-74.

16. Nattino G, Lemeshow S, Phillips G, Finazzi S, Bertolini G. Assessing the Calibration of Dichotomous Outcome Models with the Calibration Belt. The Stata Journal. 2017;17(4):1003-14.

17. Nattino G, Finazzi S, Bertolini G. Comments on 'Graphical assessment of internal and external calibration of logistic regression models by using loess smoothers' by Peter C. Austin and Ewout W. Steyerberg. Statistics in medicine. 2014;33(15):2696-8.

18. Steyerberg EW, Vickers AJ, Cook NR, Gerds T, Gonen M, Obuchowski N, et al. Assessing the performance of prediction models: a framework for traditional and novel measures. Epidemiology (Cambridge, Mass). 2010;21(1):128-38.

19. McKelvey RD, Zavoina W. A statistical model for the analysis of ordinal level dependent variables. The Journal of Mathematical Sociology. 1975;4(1):103-20.

20. Töllner U. Early diagnosis of septicemia in the newborn. European journal of pediatrics. 1982;138(4):331-7.

21. Fleiss N, Coggins SA, Lewis AN, Zeigler A, Cooksey KE, Walker LA, et al. Evaluation of the Neonatal Sequential Organ Failure Assessment and Mortality Risk in Preterm Infants With Late-Onset Infection. JAMA Network Open. 2021;4(2):e2036518-e.

22. Hanley JA, McNeil BJ. A method of comparing the areas under receiver operating characteristic curves derived from the same cases. Radiology. 1983;148(3):839-43.

23. Fitzgerald M, Saville BR, Lewis RJ. Decision curve analysis. Jama. 2015;313(4):409-10.

24. Vickers AJ, Van Calster B, Steyerberg EW. Net benefit approaches to the evaluation of prediction models, molecular markers, and diagnostic tests. BMJ (Clinical research ed). 2016;352:i6.

25. Kerr KF, Brown MD, Zhu K, Janes H. Assessing the Clinical Impact of Risk Prediction Models With Decision Curves: Guidance for Correct Interpretation and Appropriate Use. Journal of clinical oncology : official journal of the American Society of Clinical Oncology. 2016;34(21):2534-40.

26. Vickers AJ, van Calster B, Steyerberg EW. A simple, step-by-step guide to interpreting decision curve analysis. Diagnostic and Prognostic Research. 2019;3(1):18.
